# Triple-Negative Inflammatory Metaplastic Breast Cancer Presenting as a Non-Mass Lesion

**DOI:** 10.14740/jmc5342

**Published:** 2026-07-01

**Authors:** Zhi Qiao Wang, Zi Hui Huang, Jin Hui Gao, Lian Qing Hong, Cheng Yu Wu

**Affiliations:** a Department of Traditional Chinese Surgery, Nanjing University of Chinese Medicine’s Hospital of Integrated Chinese and Western Medicine, Nanjing, Jiangsu 210014, China; b Department of Pathology, Nanjing University of Chinese Medicine’s Hospital of Integrated Chinese and Western Medicine, Nanjing, Jiangsu 210014, China; c School of Traditional Chinese Medicine, Nanjing University of Chinese Medicine, Nanjing, Jiangsu 210023, China

**Keywords:** Inflammatory breast cancer, Metaplastic breast cancer, Triple-negative breast cancer, Non-mass lesions of the breast

## Abstract

Inflammatory breast cancer is exceedingly rare, especially when the pathology involves invasive carcinoma alongside metaplastic carcinoma. This disease constitutes a diagnostic and therapeutic challenge, largely due to a scarcity of documented cases in the literature. We reported a case of a female patient who presented with axillary lymphadenopathy and diffuse swelling of a single breast without a solid mass. During hospitalization, she underwent various diagnostic procedures, including lymph node fine-needle aspiration, axillary lymph node biopsy, pleural effusion puncture, magnetic resonance imaging (MRI), and positron emission tomography-computed tomography (PET-CT). Despite these extensive investigations, a definitive diagnosis could not be established. The pathological nature was ultimately clarified through a core needle biopsy of the breast lesion, which subsequently guided the clinical treatment. This case highlights the complexity and challenge of diagnosing metaplastic carcinoma within inflammatory breast cancer, as well as the necessity of performing a core needle biopsy on inflammatory lesions when no solid mass is present.

## Introduction

Breast cancer is the most common cancer worldwide and continues to contribute significantly to global cancer mortality. In 2022, it is estimated that there were approximately 2.309 million new cases of breast cancer globally, with approximately 666,000 deaths [1]. These figures underscore the substantial global burden of this disease. According to the International Agency for Research on Cancer, based on aggregated data from 185 countries, breast cancer has surpassed lung cancer as the most common cancer among women [2]. It accounts for approximately one-quarter of all female cancer cases and one-sixth of female cancer deaths, posing a serious threat to women’s health.

Inflammatory breast cancer (IBC) is a highly aggressive form of breast cancer characterized by rapid onset, high risk of metastasis, and poor clinical prognosis, accounting for 2–4% of new breast cancer cases. Owing to its high invasiveness, rapid disease progression, and tendency towards early metastasis, IBC is responsible for 8–10% of breast cancer-related deaths [3]. First described in 1814, IBC was so named due to its similarity to acute breast inflammation. Lee and Tannenbaum coined the term IBC in 1924. IBC exhibits a higher incidence among African American women [4]. Pathologically, IBC does not have a specific histological subtype and can be classified into various histological types. IBC is a rare and highly aggressive subtype of breast cancer. Although the widespread application of multidisciplinary treatment strategies, including neoadjuvant chemotherapy, surgery, radiotherapy, and targeted therapy, has improved clinical outcomes for some IBC patients in recent years, the overall prognosis remains poor. On the other hand, metaplastic breast carcinoma (MBC) is an even rarer form of breast cancer, with an incidence ranging from 0.2% to 5% among all breast cancer types. It has the worst prognosis and a notably high mortality rate [5]. Compared with triple-negative invasive breast cancer, MBC presents with larger lesions, higher invasiveness, early multiple metastases, and an even poorer prognosis [6–8]. A systematic review with individual patient data meta-analysis [9] showed that the median overall survival (OS) of MBC was 75.0 months, and median progression-free survival (PFS) was 36.0 ± 13.6 months.

In this article, we present the case of a 61-year-old female patient diagnosed with IBC combined with metaplastic carcinoma. The immunohistochemistry results indicated triple-negative breast cancer, with multiple lymph node and bone metastases present at diagnosis, suggesting an unfavorable prognosis.

## Case Report

A 61-year-old female patient presented with “swelling and pain in the left breast and left axilla for half a month,” accompanied by discomfort and pain in the costal region, lower back, and waist. Physical examination revealed diffuse swelling of the left breast with *peau d’orange* changes (Fig. 1) and nipple retraction; however, no palpable mass was detected. Multiple enlarged, moderately firm, and tender lymph nodes were palpable in the left axilla. On admission, the patient’s height was 156 cm, weight was 61 kg, and body mass index (BMI) was 25.07 kg/m^2^.

**Figure 1:**
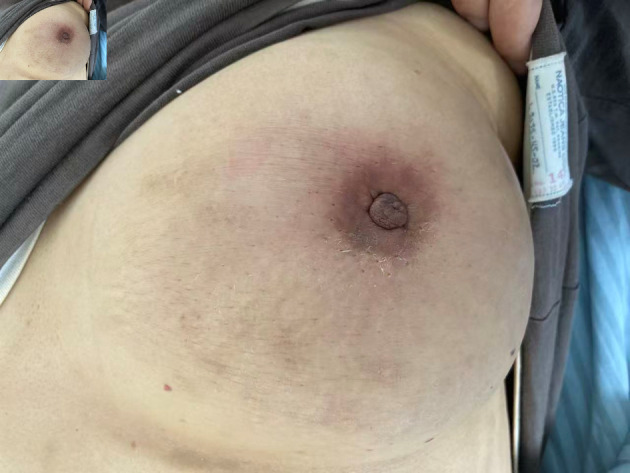
Appearance of the left breast upon patient's admission.

The patient has a history of hypertension, diabetes, coronary heart disease, chronic renal insufficiency, anxiety disorder, and other medical conditions. Blood tests indicated an inflammatory response: elevated white blood cell count (10.20 × 10^9^/L), neutrophil count (8.17 × 10^9^/L), and high-sensitivity C-reactive protein (46.42 mg/L). Tumor markers showed elevated carcinoembryonic antigen (9.12 ng/mL) and carbohydrate antigen 153 (126.4 U/mL). Ultrasound revealed structurally enlarged axillary lymph nodes (Fig. 2), but no significant mass was detected in the breast (Fig. 3), while magnetic resonance imaging (MRI) indicated abnormalities in the left breast and multiple enlarged lymph nodes (Fig. 4). Positron emission tomography-computed tomography (PET-CT) showed (Figs. 5, 6) multiple nodular soft tissue opacities in the left breast with increased fluorodeoxyglucose (FDG) metabolism; multiple lymph node metastases in the left supraclavicular and axillary regions; multiple metastases in the right anterior lobe of the liver and left adrenal gland; and multiple bone metastases throughout the body.

**Figure 2:**
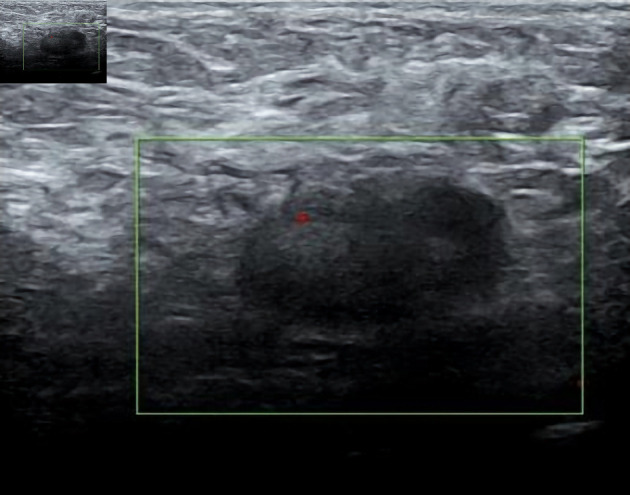
Left axillary lymph node, which appeared plump with an indistinct hilum and unclear corticomedullary differentiation.

**Figure 3:**
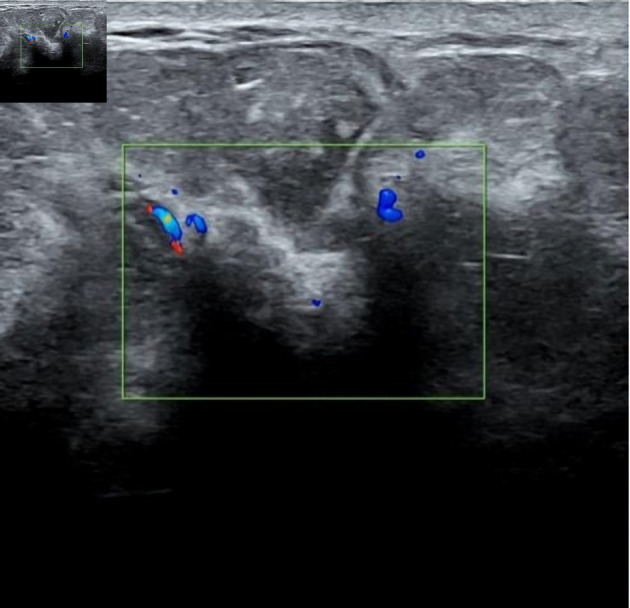
The image demonstrated edema and skin thickening in the left breast, with posterior acoustic attenuation, an indistinct glandular boundary, and disorganized echogenicity. Visualization of the glandular tissue, deep fascia, and pectoralis major muscle was poor.

**Figure 4:**
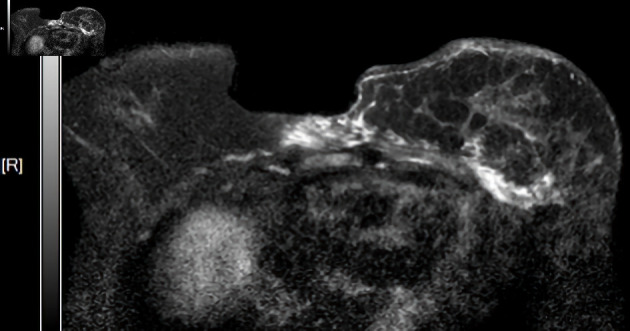
Multiple nodular T2WI hyperintense lesions were observed within the left breast, showing progressive enhancement.

**Figure 5:**
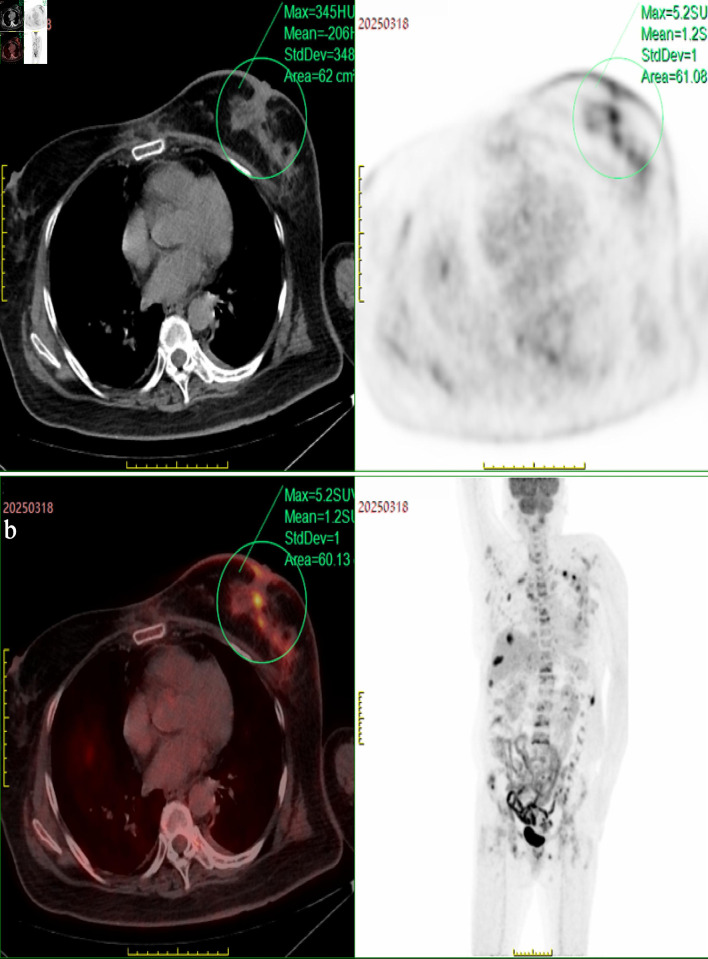
(a)The left breast was enlarged, containing multiple ill-defined nodular soft tissue lesions that demonstrated increased FDG metabolism. (b)Axial CT scan revealed the status of the lesion. The PET scan showed enhancement of the lesion. FDG: fluorodeoxyglucose; PET: positron emission tomography; CT: computed tomography.

**Figure 6:**
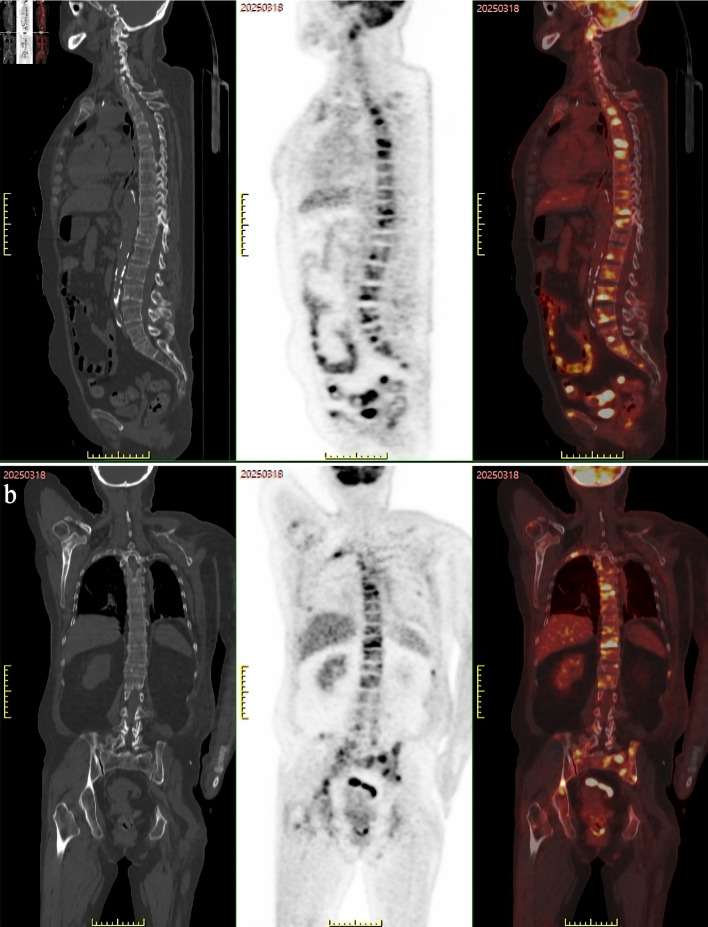
PET-CT revealed multiple osteolytic lesions with abnormally increased FDG metabolism in the bones throughout the body (axial skeleton and proximal appendicular skeleton), suggestive of widespread bone metastases. (a) Sagittal plane, showing the lesion. (b) Frontal plane, showing the lesion. PET-CT: positron emission tomography-computed tomography; FDG: fluorodeoxyglucose.

During hospitalization, the patient gradually developed pitting edema in the left upper limb. To confirm the pathology, a left axillary lymph node resection was performed, during which multiple enlarged lymph nodes measuring 1.5–2.5 cm in size were observed along the posterior edge of the left pectoralis major muscle. The lymph nodes were moderately firm, fused with each other, and some adhered tightly to the pectoralis major muscle. Two enlarged lymph nodes were completely resected. Postoperatively, the patient’s incision healed well. Based on immunohistochemical markers, our primary consideration was metastatic carcinoma. After ruling out metastases from the breast, lung, and other sites, it was determined to be metaplastic carcinoma originating from the accessory breast tissue, as shown in Figure 7. Immunomarker results were as follows: E-cadherin (E-cad, 2+), GATA-3 (2+), estrogen receptor (ER, –), progesterone receptor (PR, –), human epidermal growth factor receptor 2 (HER2, 0), Ki-67 (approximately 40% +), CK5/6 (3+), P63 (3+), P40 (3+), vimentin (3+), PAX8 (–), thyroid transcription factor 1 (TTF-1, +), CK7 (3+), Napsin A (1+), CD117 (–), CD5 (–), 34BE12 (3+).

**Figure 7:**
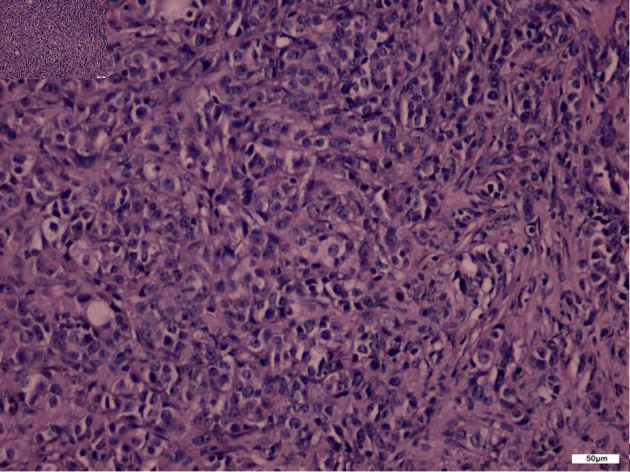
Histopathological section. Tumor cells were diffusely distributed in a mixed pattern of round, polygonal, irregular, and spindle shapes, with prominent nucleoli and significant nuclear atypia (H&E staining, × 200). H&E: hematoxylin and eosin.

Chest CT revealed a large amount of pleural effusion, and left pleural effusion aspiration was performed. The pathology report of the “left pleural effusion” indicated malignant pleural effusion, with immunohistochemical markers and clinical history suggesting metastatic breast cancer. Subsequently, a left breast core needle biopsy was performed. Further immunohistochemical staining results supported a triple-negative phenotype (ER (–), PR (–), HER2 (0)), with expression of basal-like markers such as CK5/6 and P63. Subsequent pathological examination of the left breast core needle biopsy confirmed “invasive carcinoma, partially exhibiting features of metaplastic carcinoma” (Fig. 8), consistent with the immunophenotype of the metastatic lymph nodes. Immunomarker results (Fig. 9) were as follows: E-cad (+), CK5/6 (+), P63 (+), GATA-3 (+), ER (–), PR (–), HER2 (0), vimentin (3+), 34BE12 (3+), pan-cytokeratin (CKpan, 3+), calponin (–), Ki-67 (approximately 30% +).

**Figure 8:**
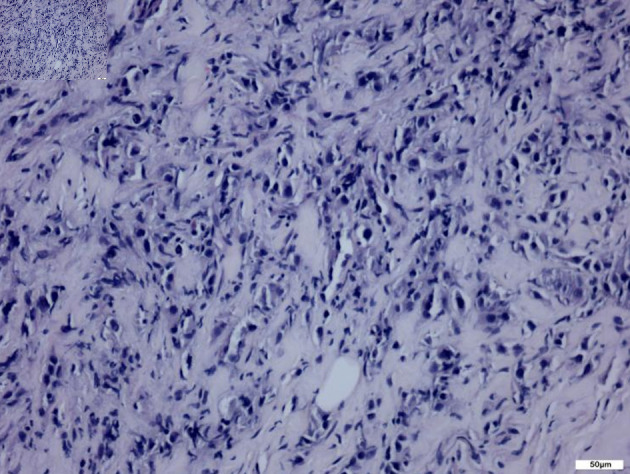
Histopathological sections (H&E staining, × 200). Round, oval, and spindle-shaped tumor cells were arranged in nests and cords. H&E: hematoxylin and eosin.

**Figure 9:**
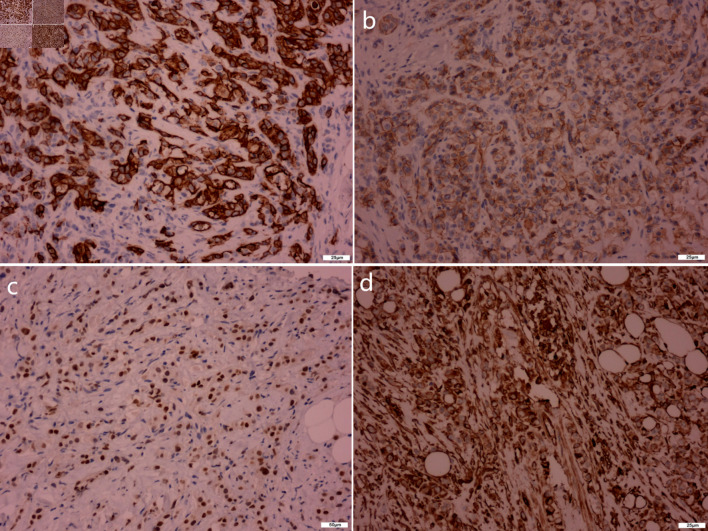
Immunohistochemical staining results (breast resection specimen, × 200). (a) Pan-cytokeratin (CKpan). (b) E-cadherin (E-cad). (c) GATA-3. (d) Vimentin. CKpan (3+), E-cad (+), GATA-3 (+), vimentin (3+), core needle biopsy revealed an epithelial neoplasm consistent with metaplastic carcinoma.

Given that the patient was at an advanced stage (stage IV) with extensive systemic metastases, the treatment focused on systemic disease control and symptom management. The patient received zoledronic acid for bone metastases and underwent pain management. During this period, unexplained recurrent high fever occurred after the placement of a chemotherapy port, and the response to anti-infective therapy and multidisciplinary consultation was unsatisfactory. An AC regimen (epirubicin 80 mg + cyclophosphamide 0.8 g) chemotherapy was ultimately attempted, but the patient showed a poor response to chemotherapy, with no reduction in tumor size.

After discharge, the patient developed severe anemia and hypoproteinemia. Due to continued disease progression, the patient eventually suffered from acute respiratory failure and passed away 2 weeks after discharge.

## Discussion

A hallmark of this case was the absence of a palpable mass, presenting radiologically as a NML. NMLs are breast lesions that lack clear boundaries on ultrasound and do not exhibit mass effects in two or more scanning planes, often appearing as patchy hypoechoic areas, duct-like hypoechoic structures, architectural distortion, or scattered and clustered microcalcifications. Early-stage breast cancer and high-risk lesions frequently manifest as NMLs [10]. In our patient, although conventional breast ultrasound detected no discrete mass, MRI revealed multiple nodular T2WI hyperintense lesions within the left breast, and PET-CT showed multiple ill-defined FDG-avid nodules accompanied by diffuse breast edema and skin thickening. These findings indicate that when clinical suspicion for IBC is high, negative conventional imaging does not exclude an underlying primary breast lesion. Furthermore, previous studies [11] have reported a significant correlation between breast tissue edema and axillary lymph node metastasis in breast cancer, which was also supported by this case. Therefore, in patients presenting with unilateral diffuse breast edema, peau d’orange change, and axillary lymphadenopathy but no palpable mass, even if ultrasound is negative, breast MRI or PET-CT should be promptly performed, followed by ultrasound-guided core needle biopsy of abnormal areas, rather than relying solely on lymph node biopsy or pleural fluid cytology.

Another typical feature of this case was the initial presentation as an axillary mass. Malignant neoplasms of the axilla encompass accessory mammary carcinoma, sarcoma, lymphoma, cutaneous mucinous carcinoma, sweat gland carcinoma, as well as metastatic deposits to the axillary lymph nodes, among other entities. Initially, it was clinically suspected that the axillary mass might be a primary malignant tumor, obstructing lymphatic drainage and leading to left breast edema with a *peau d’orange* appearance. As the condition progressed, upper limb edema developed. Following a biopsy of the axillary lymph nodes, the pathological diagnosis indicated poorly differentiated carcinoma, with the possibility of metaplastic carcinoma originating from accessory breast tissue not ruled out. Consequently, it remains unclear whether the axillary mass is primary or metastatic. A subsequent PET-CT scan revealed multiple lymph node and bone metastases, as well as pleural effusion. To further clarify the diagnosis, an ultrasound-guided core needle biopsy of the breast was performed, with multiple samples taken from the hypoechoic areas. The results confirmed invasive breast carcinoma combined with metaplastic carcinoma. This highlights the importance of considering the possibility of metastatic origin rather than a primary tumor when diagnosing metaplastic tumors and underscores the need to investigate potential lesions in other areas during the diagnostic process.

A notable diagnostic ambiguity in this case arose from the axillary lymph node biopsy, which suggested metaplastic carcinoma possibly originating from accessory breast tissue. However, subsequent breast core needle biopsy confirmed invasive carcinoma with metaplastic features within the left breast parenchyma, with an identical immunophenotype (CK5/6+, P63+, vimentin+, triple-negative) to the axillary metastasis. This apparent contradiction requires critical resolution. Several lines of evidence support a breast primary origin. First, imaging findings were highly suggestive of a breast primary. PET-CT revealed multiple ill-defined FDG-avid lesions within the left breast parenchyma, consistent with a non-mass-like primary tumor rather than inflammatory changes. MRI also showed multiple nodular lesions with progressive enhancement within the left breast. Second, epidemiologic evidence argues against primary accessory breast carcinoma, which is exceptionally rare, accounting for < 1% of all breast cancers, and typically presents as an isolated axillary mass without ipsilateral parenchymal involvement or inflammatory changes. Third, pathophysiologic evidence also favors a primary breast origin. The diffuse breast edema and *peau d’orange* change can be explained by tumor emboli in dermal lymphatics from a breast primary—a hallmark histopathologic feature of IBC. An isolated accessory breast primary would not suffice to cause diffuse edema of the entire breast. We therefore conclude that the axillary finding most likely represents metastatic spread from an occult breast primary, with the accessory breast tissue being an incidental benign finding. This interpretation resolves the apparent contradiction and aligns with the final integrated clinicopathological diagnosis.

Literature indicates that MBC can be histopathologically classified as monophasic (containing only one metaplastic component) or biphasic (containing two or more components). In this case, the pathological findings revealed both squamous cell carcinoma and adenocarcinoma, categorizing it as biphasic MBC, which is relatively rare. The co-occurrence of IBC and MBC is even rarer. In literature review, we identified only two relevant studies [12, 13], and no tumor-related deaths were reported during the follow-up periods in those cases, which contrasts with the prognosis of the patient in this case report. The patient’s survival time after diagnosis was less than 1 month, which may be associated with the following factors. First, the tumor exhibited a special pathological type. Immunophenotypically, most MBC cases are triple-negative, meaning they test negative for ER, PR, and HER2, with HER2 overexpression observed in only 15% of cases [6]. The patient had metaplastic carcinoma combined with inflammatory and triple-negative features. Studies [14–16] on IBC patients have shown that the triple-negative subtype is associated with the poorest survival prognosis. The mixed subtype has a worse prognosis than other histological subtypes [17]. Second, this unfavorable prognosis may be related to aggressive biological behavior. The unique pathological type is underpinned by highly invasive biological behavior. Research suggests that, compared to common invasive breast carcinomas of no special type, MBC exhibits distinct gene expression patterns: genes related to cell motility, migration, and extracellular matrix production are significantly upregulated, while genes responsible for intercellular junctions and adhesion are downregulated. These alterations in gene expression may explain the heightened invasiveness and migratory capacity of metaplastic carcinoma [18, 19]. Immunohistochemical and ultrastructural studies further indicate [19–22] that MBC originates from multipotent undifferentiated cells, which directly correlates with its biological characteristics. Compared to conventional breast cancer, IBC is more prone to vascular and lymphatic invasion, likely due to the synergistic effects of adhesion molecules, vascular growth factors, and protein factors released by tumor cells [23]. The tumor was located in the left breast, which has been associated with a poorer prognosis in previous studies [24]. Additionally, the family reported that the patient had experienced lower back pain for several months prior to admission, suggesting the possible presence of multiple bone metastases. This indicates a potentially prolonged latent disease course, and the patient’s refusal of relevant examinations may have contributed to the delayed diagnosis and treatment. Furthermore, the patient’s multiple comorbidities, persistent fever, advanced cancer-related cachexia, and hypercoagulable state collectively accelerated the disease progression.

This case underscores the core value of multidisciplinary collaboration in the diagnosis and treatment of complex cases. Through multidisciplinary discussions involving radiology, pathology, medical oncology, breast surgery, and interventional radiology, a definitive diagnosis was ultimately established, and an individualized treatment plan was developed. This process ensured that the patient received the optimal treatment strategy, maintained continuity and coordination of care, and minimized the risk of misdiagnosis or inappropriate management.

The main limitations of this case include: (1) As a single case report, the findings cannot be generalized to all patients with IBC or MBC; (2) Due to rapid disease progression and comorbidities, the patient did not receive standardized neoadjuvant chemotherapy or targeted therapy, precluding assessment of their efficacy in this specific subtype; (3) Autopsy was not performed, preventing confirmation of all metastatic sites and the presence of other occult lesions.

### Conclusions

Clinicians must maintain a high index of suspicion when encountering patients with unexplained axillary lymphadenopathy, even in the absence of a palpable breast mass or characteristic findings on conventional imaging. The possibility of rare yet highly aggressive malignancies, such as inflammatory breast carcinoma or MBC, should be considered. To prevent diagnostic delay, immediate initiation of a rapid and comprehensive multidisciplinary assessment is imperative. This evaluation must include pathological examination of the lymph node and systemic imaging.

The combination of advanced imaging and tissue biopsy is crucial to avoid diagnostic delays and optimize treatment. Additionally, we emphasize the necessity of a multidisciplinary consultation model, which provides more scientific, rational, and precise diagnostic and treatment plans for patients with complex conditions. This approach represents the core of high-quality medical care and is an inevitable trend in the future of medicine.

## Data Availability

Any inquiries regarding supporting data availability of this study should be directed to the corresponding author.

## Abbreviations

IBC: inflammatory breast cancer
MBC: metaplastic breast carcinoma
OS: overall survival
PFS: progression-free survival
NML: non-mass lesion

## References

[1] Bray F, Laversanne M, Sung H, Ferlay J, Siegel RL, Soerjomataram I, Jemal A (2024). Global cancer statistics 2022: GLOBOCAN estimates of incidence and mortality worldwide for 36 cancers in 185 countries. CA Cancer J Clin.

[2] Sung H, Ferlay J, Siegel RL, Laversanne M, Soerjomataram I, Jemal A, Bray F (2021). Global cancer statistics 2020: GLOBOCAN estimates of incidence and mortality worldwide for 36 cancers in 185 countries. CA Cancer J Clin.

[3] Zhu S, Zheng Z, Hu W, Lei C (2023). Conditional cancer-specific survival for inflammatory breast cancer: analysis of SEER, 2010 to 2016. Clin Breast Cancer.

[4] Bertucci F, Ueno NT, Finetti P, Vermeulen P, Lucci A, Robertson FM, Marsan M (2014). Gene expression profiles of inflammatory breast cancer: correlation with response to neoadjuvant chemotherapy and metastasis-free survival. Ann Oncol.

[5] Reddy TP, Rosato RR, Li X, Moulder S, Piwnica-Worms H, Chang JC (2020). A comprehensive overview of metaplastic breast cancer: clinical features and molecular aberrations. Breast Cancer Res.

[6] Lee H, Jung SY, Ro JY, Kwon Y, Sohn JH, Park IH, Lee KS (2012). Metaplastic breast cancer: clinicopathological features and its prognosis. J Clin Pathol.

[7] El Zein D, Hughes M, Kumar S, Peng X, Oyasiji T, Jabbour H, Khoury T (2017). Metaplastic carcinoma of the breast is more aggressive than triple-negative breast cancer: a study from a single institution and review of literature. Clin Breast Cancer.

[8] Zhang Y, Lv F, Yang Y, Qian X, Lang R, Fan Y, Liu F (2015). Clinicopathological features and prognosis of metaplastic breast carcinoma: experience of a major Chinese cancer center. PLoS One.

[9] Hettwer CC, Wurschi GW, Pietschmann K (2026). A systematic review with individual patient data meta-analysis on characteristics and outcomes of patients with metaplastic breast carcinoma. Clin Breast Cancer.

[10] Zhang J, Cai L, Pan X, Chen L, Chen M, Yan D, Liu J (2022). Comparison and risk factors analysis of multiple breast cancer screening methods in the evaluation of breast non-mass-like lesions. BMC Med Imaging.

[11] Deniz Altintas D, Esen Icten G, Taskin F, Uras C (2025). Relationships between breast edema and axillary lymph node metastasis in breast cancer. Diagnostics (Basel).

[12] Kuo SH, Chen CL, Huang CS, Cheng AL (2000). Metaplastic carcinoma of the breast: analysis of eight Asian patients with special emphasis on two unusual cases presenting with inflammatory-type breast cancer. Anticancer Res.

[13] Johnson TL, Kini SR (1996). Metaplastic breast carcinoma: a cytohistologic and clinical study of 10 cases. Diagn Cytopathol.

[14] Liu J, Chen K, Jiang W, Mao K, Li S, Kim MJ, Liu Q (2017). Chemotherapy response and survival of inflammatory breast cancer by hormone receptor- and HER2-defined molecular subtypes approximation: an analysis from the National Cancer Database. J Cancer Res Clin Oncol.

[15] Diao JD, Ma LX, Sun MY, Wu CJ, Wang LJ, Liu YL, Yang YJ (2019). Construction and validation of a nomogram to predict overall survival in patients with inflammatory breast cancer. Cancer Med.

[16] Xu F, Yang J, Han D, Huang Q, Li C, Zheng S, Wang H (2021). Nomograms for estimating cause-specific death rates of patients with inflammatory breast cancer: a competing-risks analysis. Technol Cancer Res Treat.

[17] Hu J, Lang R, Zhao W, Jia Y, Tong Z, Shi Y (2023). The mixed subtype has a worse prognosis than other histological subtypes: a retrospective analysis of 217 patients with metaplastic breast cancer. Breast Cancer Res Treat.

[18] Kline TS, Kline IK (1990). Metaplastic carcinoma of the breast—diagnosis by aspiration biopsy cytology: report of two cases and literature review. Diagn Cytopathol.

[19] Silverman JF, Feldman PS, Covell JL, Frable WJ (1987). Fine needle aspiration cytology of neoplasms metastatic to the breast. Acta Cytol.

[20] Jebsen PW, Hagmar BM, Nesland JM (1991). Metaplastic breast carcinoma. A diagnostic problem in fine needle aspiration biopsy. Acta Cytol.

[21] Tavassoli FA Classification of metaplastic carcinomas of the breast. Pathol Annu. 1992;27(Pt.

[22] Bataillon G, Fuhrmann L, Girard E, Menet E, Lae M, Capovilla M, Treilleux I (2018). High rate of PIK3CA mutations but no TP53 mutations in low-grade adenosquamous carcinoma of the breast. Histopathology.

[23] Raghav K, French JT, Ueno NT, Lei X, Krishnamurthy S, Reuben JM, Valero V (2016). Inflammatory breast cancer: a distinct clinicopathological entity transcending histological distinction. PLoS One.

[24] Chen SY, Gao Y (2024). Analysis of prognostic factors for triple-negative inflammatory breast cancer using the SEER database. Chinese Journal of Clinical Research.

